# Supplementary material for: Perceptions and Practices of Oral Health Care Professionals in Preventing and Managing Childhood Obesity
Source: Nutrients. 2022 Apr 26;14(9):1809. doi: 10.3390/nu14091809 (PMC9099995; doi:10.3390/nu14091809)
Supplement: Supplementary file 1 [file nutrients-14-01809-s001.zip › nutrients-1683590-supplementary.pdf]

Supplementary Materials:

Table S1. Semi-structured interview guide.

**Topics of discussion for the interview**
**General**
1. Do you know about the common risk factor approach?
2. What do you know about the topic of oral health and obesity? Do you think oral health is important for people with obesity—why/why not?
3. Do you think there is a link between dental caries and obesity? Perio and obesity? How do you think they are related?
**Obesity screening**
1. Do you measure the height, weight, and waist of all children or just the ones who participated in HSHK?
2. What did you feel when measuring height, weight, and waist for patients? What were your experiences?
3. Did the parents say or feel anything on what has this to do with dentistry? What were their experiences?
4. How is your knowledge about obesity and BMI? What do you know? What would you like to learn?
**Obesity prevention**
1. What kind of dietary advice do you currently provide to patients? How do you think they fit in with Dietary guidelines? Any discrepancies?
2. Do you think information about healthy eating provided in dentistry would be enough to help people make changes to their diet? If no, what more dietary advice do you think could be included for future?
3. Do you think OHCPs should give any other advice apart from diet to people with obesity? If so, what, can you be specific? (e.g., physical activity, sedentary behaviour) How do you think this advice could be given to patients? Did you give any of this as part of HSHK?
4. What do you think is the best mode of giving advice to patients? (e.g., phone messages, apps, clinic based, etc.)
**Obesity management**
1. Do you feel it would be appropriate for OHCPs to speak with patients about overweight and obesity? Do you think families are receptive to you undertaking the role on obesity? Child versus adult patients?
2. In your opinion do OHCPs have important role to promote healthy eating and healthy weight in routine care? If no, who would be someone that patients might approach about concerns for overweight and obesity? (e.g., GP, Paediatrician, Family Health Nurse, Go for Fun, Dietitian, etc.)
3. What other experiences or knowledge do you have on healthy eating practices or obesity in dentistry?
4. How do you think OHCPs could manage people with overweight and obesity? (e.g., referral)
**Barriers**
1. How do you think you could help patients achieving their healthy weight goals?
2. What do you think are the barriers to promoting healthy eating and weight counselling to people with obesity? From patients' perspective and care providers.
3. How do you think we could address these barriers?
4. Who do you think should pay? (e.g., payment, co-payment, Government, personal)
5. Is time an issue? How long do you think it will take with and without obesity screening?

Guidelines/Training/Education
1. Do you think there should be guidelines on obesity management for OHCPs? If so, what do you think should be the part of these guidelines?
2. Have you received any education/training on obesity screening, prevention, and management? If yes, where did you find this information. (e.g., undergraduate, CPD)
3. Should there be information in dental schools on obesity screening, prevention, and management? If so, what do you think could be included?
4. Do you have education resources (such as brochures, pamphlets) related to obesity, or diet or weight in your practice? If yes, what? If no, would you like resources? Any other resources?
5. Would you be interested to undertake any education/training on overweight and obesity? Will the CPD course be more attractive? If so, are you interested in an evening session, an online program, or half-day weekend seminar?
6. How do you think it is best to raise the issue with the child and the family if weight is the problem?
**Final**
1. Are there any other comments or suggestions you would like to make on this topic?
